# Facilitation of practical work in Natural Sciences: experiences and approaches of junior teachers

**DOI:** 10.12688/f1000research.138137.2

**Published:** 2024-08-30

**Authors:** Lettah Sikhosana, Khutso Charles Mogale

**Affiliations:** 1 Department of Science and Technology Education, College of Education, University of South Africa, Pretoria, Gauteng, 0002, South Africa; 2 Department of Science and Technology Education, College of Education, University of South Africa, Pretoria, Gauteng, 0002, South Africa

**Keywords:** Practical work, Novice teacher, Natural Sciences, Professional development, Approaches, Experiences, Pedagogical content knowledge

## Abstract

**Background:**

This study delved into the experiences and approaches employed by junior teachers in facilitating practical work in Natural Sciences during the teaching and learning process. In South Africa, Grade 7 marks the final year of primary school education, with learners typically aged between 11 to 13. The researchers noted significant challenges faced by junior Grade 7 teachers in preparing, organizing, and carrying out practical work within the context of teaching and learning in Natural Sciences.

**Methods:**

In the present study, a qualitative multiple-case study design was employed. Three grade 7 Natural Sciences junior-level teachers were purposefully selected as participants. Data was gathered through classroom observations, semi-structured interviews, and document analysis.

**Results:**

The study findings revealed that certain grade 7 junior Natural Sciences teachers found it challenging to manage practical work during teaching and learning. Some teachers also faced difficulties in maintaining discipline during practical work and demonstrations, both inside and outside the classroom. To cope with the demands of facilitating practical work in Natural Sciences teaching and learning, teachers employed various approaches, such as group work, observations, demonstrations, illustrations, projects, and inquiry-based scientific investigations.

**Conclusion:**

It is recommended that teachers should receive professional training on effectively facilitating practical work to support the new teaching and learning methods outlined in the Curriculum Assessment Policy Statement. They should also be equipped with pedagogical content knowledge to help them achieve the goals of practical work in teaching and learning Natural Sciences.

## Introduction

Starting a new career path in the field of education comes with unrealistic expectations about teaching which may result in negative and stressful experiences for teachers that are junior ( Dias-Lacy & Guirguis, 2017). Junior teachers can be best described as teachers who are in their first year of teaching with little teaching experience or no prior teaching experience ( Makoa & Segalo, 2021). Despite the efforts made by the Department of Basic Education and the Department of Higher Education and Training in South Africa to support junior teachers, challenges experienced by these teachers persist ( Esau & Maarman, 2021). Studies conducted in countries such as Turkey, Pakistan, the United States of America and Qatar revealed that junior teachers lack adequate professional support and may feel unprepared to handle academic issues and manage learner behaviour ( Ahmed *et al*., 2020; AL-Naimi *et al*., 2020; Ross-Hain, 2020; Kozikoğlu and Senemoğlu, 2018). It is imperative to acknowledge that pervasive challenges are not only confined to junior teachers within the African continent as they are also widely experienced by other teachers across the globe ( Lodge *et al*., 2018; Abdurrahman, 2016).

In the past two decades, the Department of Basic Education (DBE) in South Africa experienced a massive loss and reduction in the teacher population due to various reasons. These reasons included among others: the resignation of teachers for greener pastures, unhealthy working conditions in the teaching fraternity, especially salaries, retirement of teachers, unmanageable workloads and unsafe school environment ( South African Democratic Teachers Union [SADTU], 2022). Certain schools were left without scarce skilled teachers who were responsible for teaching gateway subjects such as Mathematics and Science. Despite the efforts made by the Department of Basic Education and other stakeholders to increase the number of experienced teachers with scarce skills, South Africa is still far from achieving this desired outcome ( Muremela *et al*., 2021). This resulted in an increase in teaching workload for the few remaining teachers in some schools and junior teachers entering the field of education.

On the other hand, in some South African schools, Natural Sciences subjects are often allocated to an unqualified teacher while the school waits for the posts to be advertised and filled by a qualified teacher, which in most cases would be junior teachers ( Ntuli *et al*., 2022). In some South African schools, scarce skilled teachers were replaced with less experienced and unqualified teachers to teach Natural Sciences ( Esau & Maarman, 2021). However, the SADTU general secretary, Mugwena Maluleke, refuted this claim by indicating that the problem was not that teachers were not qualified‚ it was that they were being made to teach the wrong subjects and misallocation of resources ( Maluleke, 2021). In 2021, the Minister of Basic Education, Angie Motshekga, revealed that there are 1575 unqualified and under-qualified teachers teaching in South African schools; with 218 of them appointed as permanent employees ( Motshekga, 2021). The Minister of Basic Education further added that “Over the years the Department has implemented various programmes, particularly, to assist un-and-under-qualified educators to improve their qualifications”.

The National Professional Diploma was implemented as an interim qualification for upgrading under-qualified teachers (Mhlaba & Rankhumise, 2022). However, inadequate attention is devoted to professional development, training, skills development, and technical support to enhance communication skills and manage the curriculum as a support mechanism for junior science teachers (Mhlaba & Rankhumise, 2022). This is similar to the Natural Sciences junior teachers who receive less attention when it comes to facilitating practical work in teaching and learning activities, as highlighted by Bantwini (2015). The Department of Basic Education ( DBE, 2011) emphasizes that the Natural Sciences curriculum aims to enable learners to explore and make meaning of natural phenomena, fostering curiosity by encouraging them to ask questions that could lead to further research and investigation. The curriculum outlines three specific aims for the Natural Sciences subject:

1. Specific Aim 1: ‘Doing science’,
2. Specific Aim 2: ‘Knowing the subject content and making connections’,
3. Specific Aim 3: ‘Understanding the uses of Science’

In this paper, the primary focus is on ‘Doing science,’ as this has significantly influenced the paper’s objective. The intention is to encourage positive attitudes among learners and to boost motivation for effective learning in the field of science education ( Okam & Zakari, 2017). This will be achieved by fostering the development of practical skills in scientific processes, alongside the fundamental conceptual understanding gained through a combination of hands-on and minds-on approaches to activity development ( Oliveira & Bonito, 2023). As such, practical work is regarded as a key and distinguishing aspect of science education that should be undertaken by learners as an essential component of high-quality science teaching ( Sshana & Abulibdeh, 2020).

Since the 18th century, teachers and researchers have examined the significance of practical work in science education ( Sshana & Abulibdeh, 2020). As a result, numerous studies have highlighted the various benefits of practical work, such as cultivating laboratory skills, enhancing scientific knowledge, and grasping scientific concepts and theories ( Schwichow, Zimmerman, Croker & Härtig, 2016). Recognizing these advantages, the Curriculum Assessment Policy Statement (CAPS) stipulated that Natural Sciences teachers should allocate 25% of their time to practical work and 75% to theoretical work each year, encompassing four recorded practical tasks annually, one per quarter, for assessment purposes ( DBE, 2012). It is important to note that the study specifically focuses on grade 7, which marks the end of primary school in South Africa, catering to learners aged 11 to 13. This paper delves into the experiences and approaches of junior grade 7 teachers in facilitating practical work in Natural Sciences teaching and learning. Furthermore, it is underpinned by the conceptual framework comprising three models: The Doing of Science model, the Performance model, and the Explanatory model, derived from Pekmez, Johnson, and Gott (2005).

### Problem statement

Engaging with inexperienced Natural Sciences teachers in grade 7 led the researchers to recognize potential issues with lesson preparation, curriculum design, and practical work. This paper was also informed by informal data collected during the annual Natural Sciences workshop, where new and less experienced Natural Sciences teachers participated. As a result, the researchers identified the necessity of investigating the methods used by novice and less experienced seventh-grade Natural Sciences teachers to conduct practical work in the classroom setting within the Lebopo circuit. The researchers noted significant challenges faced by grade 7 junior teachers in preparing, designing, and conducting practical work in Natural Sciences teaching and learning. As a result, some teachers expressed doubts about the effectiveness of incorporating practical work in science education, viewing it as ill-conceived, confusing, and unproductive ( Sshana & Abulibdeh, 2020). These challenges pose major obstacles to achieving national curriculum goals ( Du Plessis & Letshwene, 2020). Despite the importance of practical work in science education, many teachers in rural South African schools still overlook it due to misconceptions about departmental policies and a lack of understanding of the Curriculum Assessment Policy Statement ( Mogofe & Kibirige, 2023).

## Literature review

### The nature of practical work

Various kinds of practical work are prescribed in the Curriculum Assessment Policy Statement (CAPS). These include observations, scientific investigation, scientific enquiry, illustration, demonstration, experiments, projects, models, problem solving, case study, fieldwork, drawings, paintings, constructions, interviews, laboratory work, assignments ( DBE, 2015; Stoffels, 2005). These types of practical work enable the teacher to involve learners in teaching and learning process where they also get an opportunity to work in pairs or in groups while the teacher plays a role of being a complete observer or silent observer ( Monteiro *et al.*, 2021). Teachers are guided by the CAPS document which stipulates clearly the type of practical work that must be done per grade and per term in Natural Sciences subject in the senior phase ( DBE, 2015). According to DBE (2011), demonstrations, observations, models, investigations, assignments, projects, and illustrations were the most commonly prescribed strategies for facilitating practical work in grade 7. However, from one of the experiences as a Natural Sciences teacher, it was evident that demonstrations were the most preferred type of practical work and which were also highly favoured by most Natural Sciences teachers. Furthermore, they preferred to perform practical work while learners were observing and listening, the reason being that they are not good at facilitation, so they rather demonstrate than facilitate the activity.

### The purpose and significance of practical work

The goals of Natural Sciences education through effective practical work bring into perspective the scarcity of skills that are associated predominantly with the Natural Sciences in which secondary school education plays a crucial role as a preparation and initiation phase into the fields ( Department of Higher Education and Training [DHET], 2014). Shortages of critical skills in the fields of science and engineering are common in most developing countries, including South Africa. Education is one of the key drivers of the economy as it enables the accomplishment of national curriculum goals ( Nyoka *et al*., 2014). Accordingly, inquiry-based practical work is aimed at improving learner engagement and learner-centeredness which involves investigations conducted by learners in contextualized and relevant experiences ( Aubusson, 2011). Hence, learners are given opportunities to conduct investigations in ways that are scaffolded to meet the levels of their skills and knowledge ( Allchin, 2014). This shows that practical work does provide learners with insights into scientific practice and increases their interest in the science field and studies ( Millar, 2011). It also develops learners’ understanding of the logic of scientific inquiry and the nature of scientific knowledge as a curriculum that can be used as a tool for teaching about experimental design.

Practical work can be divided into four types: confirmatory, inquiry, discovery, and problem-based ( Banchi & Bell, 2008). According to the Department of Basic Education (2012), practical work includes demonstrations, observations, illustrations, investigations, projects, assignments, and case studies. This encompasses teacher demonstrations and experiments carried out by both teachers and learners, either cooperatively or individually ( Maričić, Cvjetičanin & Anđić, 2019). It is important to note that the CAPS document guides Natural Sciences teachers on the specific practical work required per grade and term ( DBE, 2012). For instance, in grade 7, demonstrations, observations, models, investigations, assignments, projects, and illustrations are commonly prescribed as practical work for Natural Sciences teachers ( DBE, 2011). The primary goal of practical work is to enhance learners’ comprehension and problem-solving skills, as well as their understanding of scientific principles, by emulating the methods used by scientists ( Sshana & Abulibdeh, 2020). This approach is intended to increase learner’s engagement and create a learner-centred environment where learners have the opportunity to conduct structured investigations according to their skill levels and knowledge ( Gholam, 2019). Situating practical work in the context of the South African Curriculum Assessment Policy Statement (CAPS).

Practical work in the context of the South African CAPS document gives practical expression to the knowledge, skills, and values worth learning in South African schools ( DBE, 2011). The Natural Sciences CAPS document is aimed at ensuring that learners acquire and apply knowledge and skills in ways that are meaningful to their own lives. Therefore, Natural Sciences teachers are expected to demonstrate adequate pedagogical content knowledge. Hence, the CAPS document promotes knowledge development in local contexts, while being sensitive to global imperatives ( DBE, 2015). The CAPS document prescribed practical work as part of the final promotional assessment which Natural Sciences teachers are compelled to implement by the three specific aims of Natural Sciences in grade 7 such as: (1) Doing science, (2) Knowing the subject content, and making connections, and (3) Understanding the uses of Science ( DBE, 2015).

### The conceptual framework and the design of the models

The paper’s conceptual framework was based on three models: The Doing of Science, the Performance, and the Explanatory models, as outlined by Pekmez, Johnson, and Gott (2005). This study delved into junior teachers’ experiences and approaches when conducting practical work in Natural Sciences. The conceptual framework served as an analytical tool for comprehending and interpreting the execution of practical work, including epistemological, ontological, and methodological aspects, as well as pedagogical methods of teaching. Table 1 presents the characteristics of movements influencing practical work.

**Table 1.** Characteristics of movements influencing practical work.

Movement			
Main characteristics	**Discovery learning**	**The process approach**	**Investigations**
	1. Learners expected to discover things for themselves	1. Motivates the identification of what scientists do and argues that this is what must be taught.	1. The approach was confounded on the focus that pupils should be thinking about what they are doing rather than simply applying the method practiced.
	2. Practical work is seen as how pupils will develop their thinking.	2. Content is not a priority but a scientific method.	2. The approach develops procedural and substantive understanding.
	3. This is a teaching method that leaves things open for discovery but also offers an opportunity for not discover them	3. This is a teaching method that focuses on skill and neglects content	3. The ultimate aim is to develop problem-solving skills.

The following models were also used to analyze and explain the thinking of the teachers about practical work.

### Application of the conceptual framework

**Figure 1.**
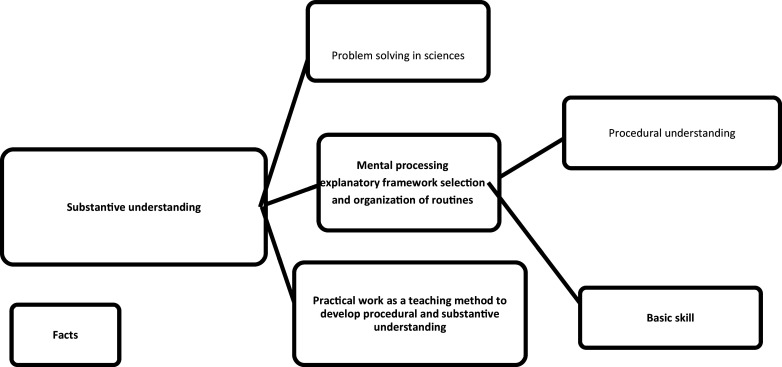
A performance model ( Pekmez *et al.*, 2005). The performance model demonstrates an investigative approach in which the teacher aims to foster both deep conceptual comprehension and procedural understanding. Practical work serves as a guiding framework, facilitating the development and application of cognitive skills. The overarching goal of this approach is to cultivate problem-solving abilities in learners.

**Figure 2.**
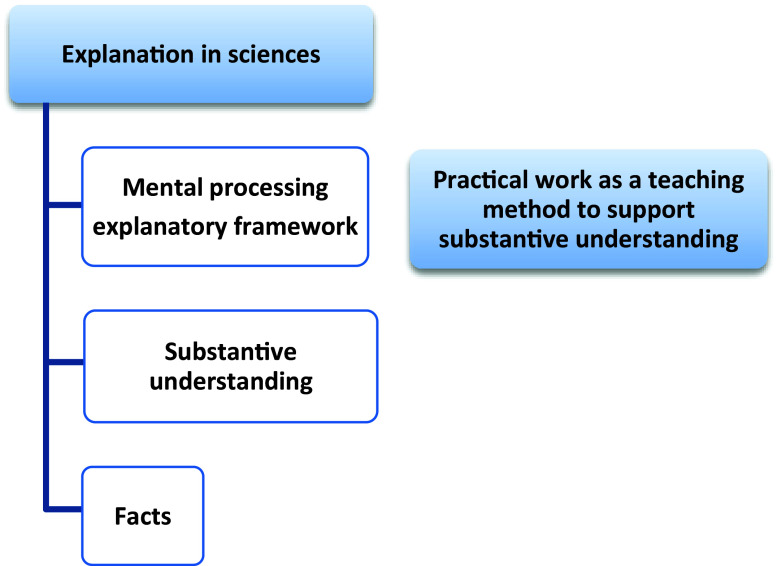
An explanatory model (Pekmez *et al.*, 2005). The explanatory model illustrated practical work as a pedagogical approach aimed at facilitating deep comprehension, while practical work was characterized as a method primarily intended for instructional purposes ( Pekmez *et al.*, 2005). Additionally, practical work served as a conceptual basis for scientific explanations.

**Figure 3.**
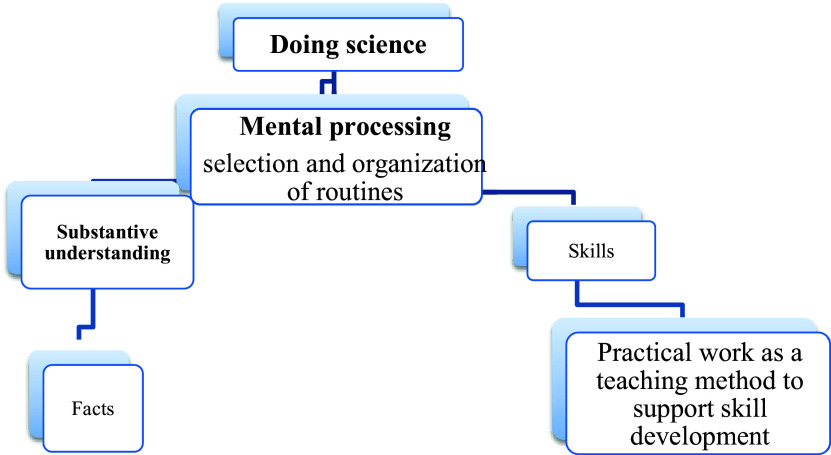
The doing of science model. The Doing of Science model was employed to assess the implementation of practical work by grade 7 Natural Sciences junior teachers in classroom settings. This approach focused on developing skills rather than solely understanding concepts, as noted by Pekmez *et al.* (2005). The teachers' perspective on practical work was also observed from a process-oriented standpoint.

In this paper, the three models mentioned above served as the conceptual framework to assess the methods through which Natural Sciences junior teachers in grade 7 conducted practical work. These models include the performance model, the explanatory model, and the doing of science model. The performance model, depicted in Figure 1, is rooted in the investigative movement approach. The teacher aims to cultivate both substantive and procedural understanding by using practical work as an explanatory framework and by selecting and organizing routines (skills) for mental processing. Our underlying objective with this approach is to cultivate problem-solving skills among learners. According to the explanation model in Figure 2, practical work is a teaching method to reinforce fundamental comprehension, while laboratory work is merely a means to this end ( Pekmez et al., 2005). Practical work is utilized as an explanatory framework for scientific explanations. The doing of science model in Figure 3 employs practical work as a teaching method to foster skills development. Pekmez et al. (2005) suggest that in this model, teachers prioritize skills over the concepts to be grasped. This model illustrates a teacher who perceives practical work from the perspective of process-oriented education.

These models have a significant impact on learners’ achievement ( Abd-El-Khalic, 2012). Teachers need to be familiar with learners’ existing misconceptions, experiences, and prior knowledge to effectively introduce new subject matter ( Chen, Sonnert, Sadler & Sunbury, 2020). Therefore, this conceptual framework serves as a reference for assessing whether teachers are knowledgeable about common misconceptions related to practical work and how they use pedagogical strategies and interactions to address any misconceptions that arise during the teaching and learning process. Additionally, we also considered the assertions made by Mudau, (2016) and, Graham, Berry, and Rowlands (2012) regarding the potential for misunderstandings to arise during instruction, whether by the teacher or the learners. Thus, our focus was not only on identifying these misconceptions but also on assessing the extent of the teacher’s pedagogical content knowledge related to practical work, and the ability to effectively impart these skills to learners during the teaching and learning of Natural Sciences as a practical subject.

### Research questions

Our main research question is ‘How do grade 7 junior teachers facilitate practical work in Natural Sciences teaching and learning?’. The following research sub-questions guided this paper: 1) What are the experiences of grade 7 junior teachers when facilitating practical work in Natural Sciences teaching and learning? 2) What pedagogical approaches are adopted by the grade 7 junior teachers when facilitating practical work in Natural Sciences teaching and learning?

In addition, the following objectives guided the study: To document grade 7 junior teachers’ experiences of facilitating practical work in Natural Sciences teaching and learning. To identify pedagogical approaches adopted by grade 7 junior teachers when facilitating practical work in Natural Sciences teaching and learning.

## Methods

### Ethics

The Unisa College of Education, South Africa, Ethics Review Committee approved this study (approval number 2017/06/14/31144187/5/MC) ( Sikhosana & Mogale, 2023d). Written informed consent was obtained from participants prior to data collection. To ensure confidentiality and anonymity, the participants were assured that all the personal information they provided will be protected. As a result, pseudonyms were used when signing the confidentiality forms and throughout the study.

### Research design

A qualitative interpretative case study was used to explore grade 7 junior teachers experiences and approaches when facilitating practical work in Natural Sciences teaching and learning. An interpretative research paradigm enabled us to understand and interpret lived experiences, views and perceptions of grade 7 junior teachers in their social context ( Kivunja & Kuyini, 2017). In our endeavour to understand how grade 7 junior Natural Sciences teachers conducted practical work, we focused on the following 2 themes:

1. Experiences of grade 7 junior teachers when facilitating practical work in Natural Sciences teaching and learning.
2. Pedagogical approaches adopted by grade 7 junior teachers when facilitating practical work in Natural Sciences teaching and learning.

### Participants

Three junior grade 7 teachers who taught Natural Sciences subject were sampled purposefully based on the purpose of this paper with the belief that each participant will provide rich and unique data (Suen *et al*., 2014). These teachers were classified as junior teachers as they had a teaching experience of less than 1 year. However, they were all qualified to teach Natural Sciences subject. Table 2 describes the demographic profile of each junior grade 7 teacher. For the purposes of this research analysis, a first year teacher will refer to an individual who entered the field of education and is teaching for the very first time in their career. As such the inexperience of a first-year teacher leads to high level of stress ( Dias-Lacy & Guirguis, 2017).

**Table 2.** Demographic profile of participants.

Grade 7 junior teachers	Subject taught	Age	Seniority	Grades taught	Qualifications
Teacher 1	Natural Sciences	23	4 Months	7-9	Bachelor of Education in Intermediate and Senior Phases
Teacher 2	Natural Sciences	26	11 Months	7-9	Bachelor of Education in Senior and Further Education and Training Phases
Teacher 3	Natural Sciences	24	6 Months	7-9	Bachelor of Education in Intermediate and Senior Phases

### Selection criteria

The drawing of the sample involved the generation of the predetermined number which were the three grade 7 Natural Sciences junior teachers at primary schools. The three teachers who participated in the study were from the Limpopo Department of Education. All the junior teachers were permanent members of staff at schools in the rural area. The following sampling criteria were used to select the participants:

• The junior teacher must have a Bachelor of Education qualification in the intermediate and senior phases.
• The junior teacher must have less than 1 year (12 months) of teaching experience.
• The junior teacher must teach Natural Sciences subject in grade 7.
• The junior teacher must have taught a topic that enhances practical work in the Natural Sciences subject.

This selection criteria played an important role in achieving the papers objective as the level of training and qualifications of the teacher might influence how the teacher’s conduct in respect to practical work ( Rogan & Grayson, 2003).

### Research setting

The setting of this study was in three primary schools in Lebopo circuit, situated around a rural area in Molepo villages, in Polokwane, Limpopo province within the Republic of South Africa. From these three primary schools, only one junior teacher who taught Natural Sciences subject in grade 7 was selected as a participant. The community’s socioeconomic status is precarious, as most residents are unemployed. These primary schools are classified as quintile 1 schools, which means they are a fee-free paying schools with limited infrastructure and resources. The Lebopo circuit is one of the five circuits in Mankweng clusters with 33 schools in Lebopo circuit of which 19 of them are primary schools. This locality has enabled the researchers to collect data easily without any difficulty because the schools were in the same vicinity and there was a viable public transport between them.

### Data collection tools

Data was collected through semi-structured interviews, lesson observations and document analysis for triangulation.

### Semi-structured interviews

Semi-structured interviews were used to collect data whereby more open questions were asked which enabled discussions with the participants rather than straightforward questions and answer ( Foley *et al*., 2021). We sought consent from the three teachers to make use of a voice recorder during the interview process to ensure that we present accurate data and word-for-word to ensure that we do not misquote them ( Sikhosana, 2022). Our interviews were conducted in two stages namely; pre-lesson observation and post-lesson observation to triangulate what was observed with what the teachers had said.

### Lesson observations

A lesson observation schedule was used to record all observational data as an essential data gathering technique in this paper. The classroom lesson observation provided us with an opportunity to have an insider perspective of how grade 7 junior teachers conducted the practical work within their settings ( Maree, 2013). The lesson observations were used to triangulate the data obtained from other techniques used such as semi-structured interviews and document analysis. With the consent from the teachers, we made used of a voice recorder during lesson observations and our lesson observation schedule to record our findings and ensure the accuracy of collected data.

During term 1, we observed Teacher 1 teaching the energy and change topic, in term 2 Teacher 2 was observed teaching the planet earth and beyond topic, and Teacher 3 was observed teaching life and living topic which was facilitated in term 3 as stipulated in the CAPS document. This was done with an aim of having an in-depth understanding on how each junior teacher facilitated practical work in the topic that they were teaching. We engaged in pre-lesson observations as well as post-lesson observations which occurred during the Natural Sciences lesson.

### Document analysis

The CAPS was used as a document to be analysed for the nature of this paper. The CAPS document was chosen as it provides valuable information that could assist grade 7 junior Natural Sciences teachers to understand how to conduct practical work, which practical work is applicable to grade 7 learners and how many times should the practical work be conducted ( DBE, 2011).

### Rigour

We ensured that the paper has internal validity by making sure that the findings of the paper are based on data collected for this paper only ( Sikhosana, 2022). To ensure that the study is credible and trustworthy, direct quotations from the participants were used when presenting all data collected ( Tawanda & Mudau, 2023). Triangulation was used to increase validity and credibility of our research findings ( Mudau & Netshivhumbe, 2022) wherein semi-structured interviews, lesson observations and document analysis were used. Pilot study conducted with one grade 7 junior Natural Sciences teacher who was not part of the main paper where both semi-structured interview, lesson observational schedules and document analysis were tested to ensure they were valid ( Ntuli *et al*., 2022).

### Data analysis

The data that was collected from three grade 7 junior Natural Sciences teachers was analysed and interpreted separately. Audio-recorded semi-structure interviews and lesson observation were transcribed verbatim by researchers to a word document ( Mudau & Netsivhumbe, 2022). While the official CAPS document was used to as a guideline to indicate how practical work must be conducted in grade 7 Natural Sciences subject ( DBE, 2015). The researchers did not correct participants’ grammatical errors to ensure that data collected from these teachers was presented accordingly and does not lose its original meaning. We analysed collected data interpretively by synthesizing, categorizing, and organizing data into themes ( Nowell *et al*., 2017). Only data relevant to the themes were considered and assisted researchers in answering research questions and achieving the aim of this study. A typology approach was used for the process of data analysis. After collecting data, we organized the data into themes. The papers research questions, aim and objectives were used to formulate the themes. We did this by immersing ourselves in the data reading to understand the whole set of data.

## Results and discussions

In this section we presented the results of three junior grade 7 Natural Sciences teachers separately. Our intentions were not to compare the cases but rather to comprehend them within their contexts which were diverse. Each case will be presented as Teacher 1, Teacher 2 and Teacher 3. The following 2 themes which assisted the researchers in answering the research questions and achieving the aim of this paper were used:

1. Experiences of grade 7 junior teachers when facilitating practical work in Natural Sciences teaching and learning.
2. Pedagogical approaches adopted by grade 7 junior teachers when facilitating practical work in Natural Sciences teaching and learning.

### Experiences of grade 7 junior teachers when facilitating practical work in Natural Sciences teaching and learning

Practical work is regarded as hands-on or minds on practical learning opportunities by learners ( Mudau & Netshivhumbe, 2022). From the semi-structured interviews that we conducted, Teacher 1 indicated that practical work has to do with using apparatus and hands. This was evident in the statement below:

*“Practical work? When you are using the apparatus, hm … using apparatus. Ooh, they are using apparatus when they are using their hands.”-Teacher 1*

From the statement mentioned above, Teacher 1 indicated that practical work has to do with using apparatus and hands. However, during lesson observations, we noted that Teacher 1 did administer practical work and grouped learners in groups of five in preparation for a Natural Sciences practical activity. Sshana and Abulibdeh (2020) argued that practical work in school of science means laboratory-based experience where there are hands-on and minds-on practical learning opportunities. DBE (2011) substantiated that practical work must be integrated with theory to strengthen the concepts being taught, which was not the case with Teacher 1, as they had inadequate understanding of practical work. Wellington and Ireson (2012) indicated that practical work is recognised as an essential part of school of sciences teaching and learning. However, that was not the case with Teacher 2 as he defined practical work as:

*“Mmhhhh … I think practical work is just investigation to find a solution to a given problem. It is when the thing that learners are going to do practical work by themselves to find the possible solution.”-Teacher 2*

From the lesson observations, we noted that Teacher 2 used the term ‘practical work’ to refer to as any teaching and learning activity which at some point involved the learners in observing or manipulating the objects and materials that they were studying. Said (2014) also attested to the fact that practical work is a learning experience in which learners interact with materials or secondary sources of data to observe and understand the natural world. Furthermore, practical work involves all kinds of learning activities in science which provokes learners to handle and observe real objects or materials ( Sshana & Abulibdeh, 2020). The observation or manipulation of objects can take place in a school laboratory but could also occurred in an out-of-school setting, such as the learners’ home or in the field ( Barendsen & Henze, 2019). This was a clear indication that Teacher 2 does not have an idea of what practical work is all about.

During the semi-structured interviews, Teacher 3 defined practical work as a teaching and learning tool of which assists learners to understand better the learning content as they are easily able to relate and put theory into practice. Teacher 3 further defined practical work as activities which involves the hands-on activities together with the minds of the learners. This was evident in the statement below

*“Practical work hmm … what I do understand most about practical work? is that you do things practically so, you display concrete things and not in a theoretical way. You must do it in a practical way so that the learners understand it in a better way, doing practical in your own hands.”-Teacher 3*

Constaninou and Fotou (2020) defined practical work as a teaching and learning transaction in which learners are given ample opportunities to practise the processes of investigation. This was similar to Teacher 3 who defined practical work as a powerful method of learning, observing and practicing hands-on activities in science. To put this in practice, Teacher 3 conducted a lesson which focused on energy and change as stipulated in the CAPS document. During this practical lesson, Teacher 3 rolled an apple across the floor and asked learners to feel the apple and also roll it on the floor; to gain a closer observation. Teacher 3 conducted this activity with an aim of developing observation skills of the learners as stated in the statement below:

*“Do that in practice using your own hands and minds for developmental purpose which prepares learners for the real world outside the school premises.”-Teacher 3*

The handling and using of materials is a key aspect to practical activities for students ( Kibga *et al*., 2021). As Teacher 3 rolled an apple on the floor, learners were also given chance to roll it by themselves and observed the movement of an apple until it stopped. This practical activity was an essential part in the lesson that Teacher 3 taught, as it enabled practical work during teaching and learning ( Wellington & Ireson, 2012). Agreeing to that, Teacher 3 displayed an adequate understanding of what practical work entailed.

Darling-Hammond *et al*. (2020) stated that teachers viewed practical work as illustrations and consolidations of understanding science concepts. From the lesson observations it was noted that Teacher 1 also viewed practical work as illustrations. Which focuses on developing learners’ understanding of the nature of science whereby learners are able to understand much of the concepts taught ( Sshana & Abulibdeh, 2020). Furthermore, Teacher 1 viewed practical work as a holistic measure to incorporate theory into practice for a better understanding of the learning content knowledge. During lesson observations, it was noted that Teacher 1 focused much more on the content, rather than developing the process and scientific skills of the learners. These are often learner-centered, constructivist approaches that include analysis and discussion of social, technological, and environmental issues.

Motlhabane (2013) indicated that teachers doubt the usefulness of practical work in relation to content comprehension. Therefore, it will be futile exercise to attempt to assist teachers without first ascertaining their perceptions on practical work. Teacher 2 viewed practical work as a process approach which was used to develop procedural skills and substantive understanding of the learners. Even if learners were in small group, this was to say that in a group of five learners, when given a task to perform, there were still those to observe while others or a group leader is performing the practical work because they have different roles to play in a group. Teacher’s views of teaching and learning science often have a pervasive influence on their classroom practices ( Bantwini, 2015). Teacher 1 liked to demonstrate first to the learners the practical work especially if the theme or the topic was new to the learners. From the semi-structured interviews, we can also infer that Teacher 2 also viewed practical work as a vehicle through which learners can ascertain their different types of skills which includes among others; the scientific inquiry skill, procedural understating skills, substantive understanding skills, creative and artistic skills, independent thinking skills, and investigative skills. This was evident in the statement below when he referred to the practical work as a teaching tool to enhance teaching and learning:

*“Mh* … *I think practical work is just investigation to find a solution to a given problem. No, practical I think is the thing that learners are going to do it themselves to find the possible solution.”-Teacher 2*

Teacher 2 viewed practical work as an important aspect in science development. They believed that practical work is the key way for students to learn, if done accurately and appropriately. Teacher 2 also believed in illustrations and consolidation through cooperative learning. This was evident during lesson observations when Teacher 2 grouped learners to use the information collected from the practical work and represent it in a graph on the record sheet provided. They viewed practical work as the investigation of the given problem in order to find answers or solution to the problem or question; this was evident in the statement below:

*“Practical work can be seen as investigating a given problem or question to find a solution.: -Teacher 2*

This is also emphasized by literature whereby it is indicated that practical work is perceived as a prominent and distinctive feature of science education by science teachers which enables learners to enrich their knowledge while developing a better understanding of science education ( Darling-Hammond *et al*., 2020).

Teacher 3 viewed practical work in Natural Sciences as a way of making the subject enjoyable and as a way of developing learners’ experimental skill ( Ramnarain, 2014). This was evident in the statement below:

*“From the food that they have ate, then that is the theoretical. And there after I asked one learner to move around so for that particular learner to be able to move around it is because he has the energy in him. This energy is kinetic energy (movement) but before kinetic energy the boy was at rest possessing stored energy which is called potential energy.”-Teacher 3*

Dudu and Vhurumuku (2012) also attested to the fact that teachers’ focus when teaching science was on the mastery of the subject matter than practical work. For many teachers, practical work provides the evidence for existing scientific knowledge and developing new knowledge ( Ntuli *et al*., 2022). Traditionally, teachers viewed the use of practical work as illustrations and consolidations of the understanding of science concepts. Teacher 3 believed that learners must be involved in doing all the theory into practice because she believed learners learn more when they see, hear, touch, and they will never forget. Teacher 3 shown the urge that theory must be put into practice because learners learn easily and enjoy when they are doing things by themselves. From the lesson observations, we saw how they asked learners to throw the ball in the air and observe what was happening and why. The learners observed and reported back in the classroom. According to Maree (2013), observations plays an important role in collecting data as it provides researchers with an insider perspective of the participants behaviors in their natural setting. In this regard, lesson observations helped learners to develop observation skills during Teacher 3 lesson of the gravitational force. Teacher 3 also viewed practical work as integration of theory into practice to strengthen the concepts being taught ( DBE, 2011). They believed that the learners needed:

*“To hear, see, touch the materials they are working with or investigating so as to enable them to respond more informative when recording their finding from the given practical tasks in a classroom context which will relate to the real day to day outside world experiences they would encounter in life.”-Teacher 3*

### Pedagogical approaches adopted by grade 7 junior teachers when facilitating practical work in Natural Sciences teaching and learning

Shulman (1986) argued that the focus of pedagogical content knowledge (PCK) is not only on the knowledge of a specific theme by a Natural Sciences teacher but also on their behaviours, reasons, and actions towards the subject content. Furthermore, Shulman (1986) stated that a presentation of PCK is simply a combination of the three facets which include: pedagogy; content; and the context. Therefore, the teacher should also know which parts of learning can be assessed and which assessment techniques should be applied ( Monteiro *et al.,* 2021). From the lesson observations, Teacher 1 conducted practical work activity which involved learners who took part in the demonstration and learners who simply observed when the demonstration was taking place. This activity gave learners an opportunity to answer questions which were posed by Teacher 1 as they were busy demonstrating and observing the procedures of cooking soup with an aim of understanding the input and output energy as stated below:

*“What are the input and output? … the input energy is the electrical potential energy and the output is the heat energy are we together?).”-Teacher 1*

However, from the lesson observations, not all learners were able to identify the input and output energy when cooking a soup as not all of them were involved in the observation that occurred. As a result, learners were unable to recognise and note what was observed as they were only engaged through oral questioning to attract and arouse their interest in the practical work that was done ( Neubauer *et al*., 2019).

Teacher 1 did practical work within the parameters of an explanatory model which it was explained and illustrated more during teaching. However, that was not effective as learners failed to answer the questions that Teacher 1 asked. This was evident in the statement below:

*“From what you have observed, how was the electrical potential energy transferred into heat energy? Come on! guys! electrical potential energy is converted into heat energy when the plate of the stove started to become hot and eventually got red, and we said you cannot create energy right? the energy can be converted, so that electrical potential energy is converted too.”-Teacher 1*

From the classroom observation, it was evident that Teacher 2 preferred conducting practical work as demonstrations. They presented various reasons for conducting practical work either as a group work or by demonstrating. Teacher 2 has a rich pedagogical content knowledge (PCK) of which their understanding of the ways for the transformation of disciplinary content into forms that are comprehensible and accessible to students ( Shulman, 1986). Amongst others, they indicated that the amount of equipment as well as the disciplinary issues influences whether if it will be group work or demonstration. However, besides all the reasons Teacher 2 gave, the data showed that they generally preferred conducting demonstrations. The teacher’s PCK helps the learners to develop knowledge of basic science procedures and utilise them to engage in the science content (Miller, 2011). This was the evident that from the interviews as mentioned by Teacher 2 in the statement below:

*“Eyah …. hei!, mmh …., so … sometimes, eh … they have to handle the materials that we are going to use with care because some are fragile, some of the things are poisonous, so. I usually tell them that eh. You have to handle these materials with care and if they need love, they are given love first.”-Teacher 2*

But during Teacher 2 lesson presentation, they did not make much of the learner involvement as stated from the interviews statement above. Only three learners used the apparatus and the rest observed and answered oral questions. This was evident in the statement below:

*“So, from our findings we are going to draw a bar graph on our record sheet provided to you. You know how to draw a bar graph right! So, our bar graph will have the X-axis here and the Y-axis. Then the X-axis will show us the three different types of metal whereas the Y-axis will show us the time in minutes, right! Therefore, you will draw the three bars on each metal rods verses the time taken by pins to fall. This is how you are going the represent the findings of our practical investigation on the bar graph.”- Teacher 2*

Teaching science successfully, teachers needed to have not only good content knowledge but also pedagogical content knowledge ( Shulman, 1986). Teacher 2’s pedagogical strategy was demonstrative and investigative. This is because the student-centred pedagogy verified by the central government in China is important to learners compared to the teacher centred approach ( Wang, 2011). From the interviews, Teacher 2 also stated that in subjects like Natural Sciences, theory must be put into practice, hence the involvement of the learners. Furthermore, Teacher 2’s PCK was a critical factor that influenced the teaching and learning ( Karisan *et al*., 2013).

*“Yah, eh … theory and practical’s are matter related because you teach them what is going to happen or the see for themselves. They need practical and see for themselves what is happening, they give reasons of what is happening when are doing the practical part of it.”-Teacher 2*

The findings from this study revealed that Teacher 2 is inclined towards a practical model of teaching. This was similar to Stoffels (2005) who indicated that some teachers preferred to conduct demonstrations than practical work during teaching and learning. The practical work conducted was affected by several factors. The large class size did not allow for the formation of groups of manageable sizes. Ten learners per group was too large a group for all learners to be directly involved with the practical work. Not all learners were therefore able to acquire skills as required from the practical work, but all learners were able to draw a bar graph representing the findings. Teacher 2 was satisfied by the availability of resources:

*“No … this one, eh besides, eh …, this lack of resource, I think yah. but it is also a problem that lack of resources because learners need to be grouped in manageable groups, and I think these groups could work more effectively, so … because of the lack of resources, we work with big groups and that is where lies a problem.”-Teacher 2*

From the classroom we observed that the lack of adequate supply of equipment did not allow for groups to be made smaller. Instead, the shortage of equipment led Teacher 2 to teach using a teacher-centred approach because learners were not divided into small groups but into too large group of learners thus preventing each learner in the group from being fully involved in the practical investigation. Moreover, the PCK is a procedure offering learners specific methodology or principle with the focus on how the learners learn together with the “context and resources” forming part of learning ( Starkey, 2012).

Teacher 3 was confined within the parameters of performance model and explanatory model ( Setiadi & Irhasyuarna, 2017). They demonstrated the rolling movement to show how the transfer of chemical potential energy to kinetic energy took place. Teacher 3 also used an explanation of what happens to an object thrown in the sky but does not remain in the sky, reason being that the object would return on the earth of because of the gravitational force. Teacher 3 had adequate content knowledge and showed competency in teaching the subject matter. According to Setiadi and Irhasyuarna (2017) the explanatory model is described as a practical work that can be used to develop an understanding of the content that is being taught. According to Hurrell (2021) teachers’ procedural understanding and knowledge is the knowing how part of teaching the subject matter or a topic. This was also showcased by Teacher 3 when they explained understand why a soccer ball cannot stay in the atmosphere forever because of the gravitational force. Practical work is also used as an explanatory framework for the explanations in science.

*“Firstly, I have to find out if they have previous knowledge about what is to transpire and if they do have that, then I take it from there then I introduce the new topic. Like let say for example when I say: did you have some breakfast? And the learners replied “yes” and then the energy, the energy … where do you get the energy from?”-Teacher 3*

From the classroom observation we can infer that Teacher 3 conducted the practical work as demonstration, and as illustrations. This was explicitly shown when Teacher 3 explained why the objects are attracted by the force of gravity when thrown in the sky. The learners were able to observe Teacher 3 throwing the apple in the sky, while doing so, Teacher 3 asked learners as to why the apple could not remain in the sky. Learners tried to answer by saying that the initial mass of every object is attracted by the earth’s gravitational force. What Teacher 3 was doing is attested by Shulman (1986) stating that the PCK of the teacher is their understanding of how to help learners to understand specific subject matter. Teacher 3 asked:

*“What type of energy is in this apple? right we said we must eat so that we can have the energy in our body. Would anyone explain what potential energy is? Yes, Potential energy is the energy stored in a system. But an apple is not a system right! Potential energy is the energy that is waiting to be released. Yes, we said potential energy is the energy that is awaiting to be released right! She has eaten the apple, then the energy has been transferred.”-Teacher 3*

Teacher 3 used practical work in a variety of formats. These formats include among others, the recipes, open-ended investigations, skills training, teacher demonstrations to promote discussion about phenomena, to raise questions, and to solve problems.

## Conclusion and recommendations

It is crucial to recognize the significance of boosting teachers’ confidence to enhance their day-to-day performance ( Sshana & Abulibdeh, 2020). The study demonstrated an effort to incorporate practical work, albeit outside the established framework. It is evident that teachers require increased confidence to effectively handle practical work, as highlighted by the study’s findings. While the paper’s representation of most science teachers is lacking, the results can inspire a better understanding of practical work and its application. The findings underscore the necessity of aligning the understanding of practical work with teaching practices for the successful implementation of an inquiry approach. This prompts a revaluation of the existing methods used to educate junior Natural Sciences teachers on facilitating practical work. The results of this study indicated that some junior Natural Sciences teachers at the grade 7 level struggled with conducting practical work effectively during teaching and learning sessions. Furthermore, some of these teachers found it challenging to maintain discipline both inside and outside the classroom when conducting practical work and demonstrations. To manage these demands, these teachers employed various strategies such as group work, observations, demonstrations, illustrations, projects, and inquiry-based scientific investigations. It is recommended that teachers receive professional training to enhance their ability to facilitate practical work in accordance with the Curriculum Assessment Policy Statement. Additionally, they should be equipped with pedagogical knowledge to effectively accomplish the goals and objectives of practical work in Natural Sciences.

## Data Availability

Figshare: Teacher 1, Teacher 2 And Teacher 3 consent forms.pdf, https://doi.org/10.6084/m9.figshare.23402228.v5 ( Sikhosana & Mogale, 2023a). The project contains the following underlying data:
• TEACHER 1 CONSENT FORM.pdf • TEACHER 2 CONSENT FORM.pdf • TEACHER 3 CONSENT FORM.pdf Figshare: Teacher 1, Teacher 2 and Teacher 3 interviews transcripts.pdf, https://doi.org/10.6084/m9.figshare.24352795.v1 ( Sikhosana & Mogale, 2023b). The project contains the following underlying data:
• TEACHER 1 INTERVIEW TRANSCRIPT.pdf • TEACHER 2 INTERVIEW TRANSCRIPT.pdf • TEACHER 3 INTERVIEW TRANSCRIPT.pdf Figshare: Teacher 1, Teacher 2 and Teacher 3 observation schedules.pdf, https://doi.org/10.6084/m9.figshare.24352792.v1 ( Sikhosana & Mogale, 2023c). The project contains the following underlying data:
• TEACHER 1 OBSERVATION SCHEDULE.pdf • TEACHER 2 OBSERVATION SCHEDULE.pdf • TEACHER 3 OBSERVATION SCHEDULE.pdf Figshare: SRQR checklist for ‘Facilitation of practical work in Natural Sciences: experiences and approaches of junior teachers’, https://doi.org/10.6084/m9.figshare.23404679.v2 ( Sikhosana & Mogale, 2023e). Data are available under the terms of the Creative Commons Attribution 4.0 International license (CC-BY 4.0).

## References

[ref56] Abd-El-Khalic F : Teaching with and About Nature of Science, and Science Teacher Knowledge Domains. *Sci. Educ.* 2012;22:2087–2107. 10.1007/s11191-012-9520-2

[ref1] Abdurrahman KOCA : Problems of novice teachers: Challenges vs. support. *J. Edu. Black Sea Reg.* 2016;1(2).

[ref2] Ahmed G Faizi WUN Akbar S : Challenges of Novice Teachers and Strategies to Cope at Secondary Level. *Global Regional Review, Humanity Only.* 2020;V(1):403–416. 10.31703/grr.2020(V-I).44

[ref4] Al-Naimi S Romanowski M Du X : Novice Teachers’ Challenges and Coping Strategies in Qatari Government Schools. *Int. J. Learn. Teach. Educ. Res.* 2020;19:118–142. 10.26803/ijlter.19.9.7

[ref3] Allchin D : From science studies to scientific literacy: A view from the classroom. *Sci. Educ.* 2014;23(9):1911–1932. 10.1007/s11191-013-9672-8

[ref5] Aubusson P : An Australian science curriculum: Competition, advances and retreats. *Aust. J. Educ.* 2011;55(3):229–244. 10.1177/000494411105500305

[ref57] Banchi H Bell R : The many levels of inquiry. *Sci. Child.* 2008;46(2):26–29.

[ref6] Bantwini B : Do Teachers’ Learning Styles Influence Their Classroom Practices? A Case of Primary School Natural Science Teachers from South Africa. *Int. J. Educ. Sci.* 2015;11:1–14. 10.1080/09751122.2015.11890369

[ref7] Barendsen E Henze I : Relating Teacher PCK and Teacher Practice Using Classroom Observation. *Res. Sci. Educ.* 2019;49:1141–1175. 10.1007/s11165-017-9637-z

[ref58] Chen C Sonnert G Sadler PM .: The Impact of High School Life Science Teachers’ Subject Matter Knowledge and Knowledge of Student Misconceptions on Students’ Learning. *Education.* 2020;19(1): mar9. 10.1187/cbe.19-08-0164 32108561 PMC8697650

[ref8] Constaninou M Fotou N : The Effectiveness of a Must-Have Practical Work in Tertiary Life Science Education. *Information.* 2020;11:401. Contexts. International Journal of Higher Education, 6(5). 10.5430/ijhe.v6n5p26. 10.3390/info11090401

[ref9] Darling-Hammond L Flook L Cook-Harvey C : Implications for educational practice of the science of learning and development. *Appl. Dev. Sci.* 2020;24(2):97–140. 10.1080/10888691.2018.1537791

[ref59] Department of Basic Education: *Curriculum assessment policy statement grade 7-9 natural science.* Pretoria: Department of education documents;2012.

[ref10] Department of Basic Education: *Natural sciences curriculum and assessment policy statement grade 7-9.* Pretoria: Department of Basic Education;2011.

[ref11] Department of Education: *Curriculum assessment policy statement grade 7-9 natural science.* Pretoria: department of education documents;2015.

[ref55] Department of Higher Education and Training [DHET]: Call for comments on the national scarce skills list: Top 100 occupations in demand. Government Gazette. (No. 37678);2014. Reference Source

[ref41] Dias-Lacy SL Guirguis RV : Challenges for New Teachers and Ways of Coping with Them. *Int. J. Educ. Learn.* 2017;6(3):265. Dias-Lacy & Guirguis, 2017. 10.5539/jel.v6n3p265

[ref60] Du Plessis E Letshwene JM : A reflection on identified challenges facing South African teachers. *IJTL.* 2020;15:69–91.

[ref12] Dudu WT Vhurumuku E : Teachers’ practices of inquiry when teaching investigations: A case study. *J. Sci. Teach. Educ.* 2012;23:579–600. 10.1007/s10972-012-9287-y

[ref13] Esau DE Maarman R : Re-imaging support for beginner teachers in relations to initial teacher education policy in South Africa. *S. Afr. J. Educ.* 2021;41(4):1–8. 10.15700/saje.v41n4a1906

[ref14] Foley G Timonen V Conlon C : Interviewing as a Vehicle for Theoretical Sampling in Grounded Theory. *Int. J. Qual. Methods.* 2021;20:160940692098095. 10.1177/1609406920980957

[ref61] Gholam A : Inquiry-Based Learning: Student Teachers’ Challenges and Perceptions. *Journal of Inquiry & Action Education.* 2019;10(2).

[ref62] Graham T Berry J Rowlands S : Are ‘misconceptions’ or alternative frameworks of force and motion spontaneous or formed prior to instruction? *Int. J. Math. Educ. Sci. Technol.* 2012;44:84–103. 10.1080/0020739X.2012.703333

[ref15] Hurrell DP : Conceptual knowledge OR Procedural knowledge OR Conceptual knowledge AND Procedural knowledge: Why the conjunction is important for teachers. *Aust. J. Teach. Educ.* 2021;46(2):57–71. 10.14221/ajte.2021v46n2.4

[ref16] Karışan D Şenay A Ubuz B : A SCIENCE TEACHER’S PCK IN CLASSES WITH DIFFERENT ACADEMIC SUCCESS LEVELS 2013.

[ref17] Kibga ES Sentongo J Gakuba E : Effectiveness of Hands-On Activities to Develop Chemistry Learners’ Curiosity in Community Secondary Schools in Tanzania. *J. Turk. Sci. Educ.* 2021;18(4):605–621. 10.36681/tused.2021.93

[ref18] Kivunja C Kuyini AB : Understanding and Applying Research Paradigms in Educational 2017.

[ref19] Kozikoğlu İ Senemoğlu N : Challenges Faced by NoviceBeginner Teachers: A Qualitative Analysis. *J. Qual. Res. Educ.* 2018;6:1–31. 10.14689/issn.2148-2624.1.6c3s16m

[ref54] Lodge JM Kennedy G Lockyer L : Understanding Difficulties and Resulting Confusion in Learning: An Integrative Review. *Front. Educ.* 2018;3(49). 10.3389/feduc.2018.00049

[ref53] Makoa M Segalo L : Teachers’ Experiences of Challenges of their Professional Development. *Int. J. Innov. Creativity Chang.* 2021;15(10).

[ref20] Maluleke M : South African schools have 5,139 teachers who are unqualified or under-qualified. 2021. Reference Source

[ref21] Maree K : *First Steps in Research.* Pretoria: Van schaik;2013.

[ref63] Maričić M Cvjetićanin S Anđić B. : Teacher-demonstration and student hands-on experiments in teaching integrated sciences. *J. Balt. Sci. Educ.* 2019;18(768-779):768–779. 10.33225/jbse/19.18.768

[ref37] Mhlaba RE Rankhumise MP : Mentoring novice beginner natural science teachers: a case study in the Gauteng province. *S. Afr. J. Educ.* 2022;42(1). Reference Source

[ref22] Millar R : Practical work. Osborne J Dillon J , editors. *Good practice in science teaching: What research has to say.* Maidenhead: Open University Press;2011; pp.108–134.

[ref64] Mogofe RA Kibirige I : *Factors hindering science teachers from conducting practical work in Sekhukhune district, Limpopo.* 2023.

[ref28] Monteiro V Mata L Santos NN : Assessment Conceptions and Practices: Perspectives of Primary School Teachers and Students. *Front. Educ.* 2021;6:631185. 10.3389/feduc.2021.631185

[ref29] Motlhabane A : The voice of the voiceless: Reactions on science practical work in rural disadvantaged schools. *Mediterr. J. Soc. Sci.* 2013;4(14):165–173. 10.5901/mjss.2013.v4n14p165

[ref30] Motshekga A : These are the provinces with the most unqualified teachers. 2021. Reference Source

[ref65] Mudau AV : The classroom practice diagnostic framework: A framework to diagnose teaching difficulties of a science topic. *EURASIA J. Math., Sci Tech. Educ.* 2016;12(11):2797–2815. 10.12973/eurasia.2016.02305a

[ref31] Mudau AV Netshivhumbe NP : Insights into the interaction and discourse in the senior phase natural sciences classroom. *Int. J. Res. J. Bus. Soc. Sci.* 2022;11:458–467. 10.20525/ijrbs.v11i6.1967

[ref32] Muremela G Kutame A Kapueja I : Retaining scarce skills teachers in a South African rural community: an exploration of associated issues. *African Identities.* 2021;21:743–759. 10.1080/14725843.2021.1965864

[ref33] Neubauer BE Witkop CT Varpio L : How phenomenology can help us learn from the experiences of others. *Perspect. Med. Educ.* 2019;8(2):90–97. 10.1007/s40037-019-0509-2 30953335 PMC6468135

[ref34] Nowell LS Norris JM White DE : Thematic Analysis: Striving to Meet the Trustworthiness Criteria. *Int. J. Qual. Methods.* 2017;16(1):160940691773384. 10.1177/1609406917733847

[ref35] Ntuli TG Nkanyani TE Sikhosana L : Exploring Teacher Knowledge in Natural Sciences. *Int. e Jour. Edu. Studies.* 2022;6:235–245. 10.31458/iejes.1192675

[ref36] Nyoka A DuPlooy E Henkeman S : *Reconciliation for South Africa’s education system.* Elm Magazine;2014. Reference Source

[ref67] Okam CC Zakari II : Impact of Laboratory-Based Teaching Strategy on Students’ Attitudes and Mastery of Chemistry in Katsina Metropolis, Katsina State, Nigeria. *Int. J. Innov. Res. Dev.* 2017;6(1):112.

[ref66] Oliveira H Bonito J : Practical work in science education: a systematic literature review. *Front. Educ.* 2023;8:1151641. 10.3389/feduc.2023.1151641

[ref68] Pekmez ES Johnson P Gott R : Teachers’ understanding of the nature and purpose of practical work. *Res. Sci. Technol. Educ.* 2005;23(1):323.

[ref38] Ramnarain U : Teachers’ perceptions of inquiry-based learning in urban, suburban, township and rural high schools: The context-specificity of science curriculum implementation in South Africa. *Teach. Teach. Educ.* 2014;38:65–75. 10.1016/j.tate.2013.11.003

[ref70] Rogan JM Grayson DJ : Towards a theory of curriculum implementation with particular reference to science education in developing countries. *Int. J. Sci. Educ.* 2003;25(10):1171–1204. 10.1080/09500690210145819

[ref39] Ross-Hain L : Transitions in tumultuous times: Teachers’ experiences with distance learning amidst the COVID-19 pandemic 2020. Reference Source

[ref40] Said Z : The importance of practical activities in school science: perspectives of independent school teachers in Qatari schools. 2014.

[ref69] Schwichow M Zimmerman C Croker S : What students learn from hands-on activities? *J. Res. Sci. Teach.* 2016;53:980–1002. 10.1002/tea.21320

[ref42] Setiadi I Irhasyuarna Y : Improvement of Model Student Learning Through the Content of Solutions Guided Discovery Buffer. *IOSR Journal of Research & Method in Education.* 2017;07:01–09. 10.9790/7388-0701050109

[ref43] Shulman LS : Those Who Understand: Knowledge Growth in Teaching. *Educ. Res.* 1986;15(2):4–14. 10.2307/1175860

[ref44] Sikhosana L : Clarifying the significance of instructional methodologies for environmental education integration. *Int. J. Res. Bus. Soc. Sci.* 2022;11(7):240–248. 10.20525/ijrbs.v11i7.2016

[ref26] Sikhosana L Mogale KC : Ethics clearance certificate.pdf. figshare.Dataset.2023d. 10.6084/m9.figshare.23992179.v1

[ref27] Sikhosana L Mogale KC : Standards for Reporting Qualitative Research (SRQR). figshare.Dataset.2023e. 10.6084/m9.figshare.23404679.v2

[ref23] Sikhosana L Mogale KC : Teacher 1, Teacher 2 and Teacher 3 consent forms.pdf. figshare.Dataset.2023a. 10.6084/m9.figshare.23402228.v5

[ref24] Sikhosana L Mogale KC : Teacher 1, Teacher 2 and Teacher 3 Interview Transcripts. figshare.Dataset.2023b. 10.6084/m9.figshare.24352795.v1

[ref25] Sikhosana L Mogale KC : Teacher 1, Teacher 2 and Teacher 3 Observation Schedule. figshare.Dataset.2023c. 10.6084/m9.figshare.24352792.v1

[ref45] South African Democratic Teachers’ Union: SADTU says schools are no longer safe for teachers and learners. 2022. Reference Source

[ref46] Sshana ZJ Abulibdeh ES : Science practical work and its impact on students’ science achievement. *J. Technol. Sci. Educ.* 2020;10(2):199–215. 10.3926/jotse.888

[ref47] Starkey L : Teaching and Learning in the Digital Age. 2012. 10.4324/9780203117422

[ref48] Stoffels NT : “There is a worksheet to be followed”: A case study of a science teacher’s use of learning support texts for practical work. *Afr. J. Res. Sci. Math. Technol. Educ.* 2005;9(2):147–157. 10.1080/10288457.2005.10740585

[ref52] Suen L Huang H Lee H : A Comparison of Convenience Sampling and Purposive Sampling. J. Nurs. 2014;61:105–111. 10.6224/JN.61.3.105 24899564

[ref49] Tawanda T Mudau AV : The influence of indigenous knowledge on chemistry metacognition. *F1000Res.* 2023;12:589. 10.12688/f1000research.131685.1 38778813 PMC11109572

[ref50] Wang D : The dilemma of time: Student-centered teaching in the rural classroom in China. *Teach. Teach. Educ.* 2011;27:157–164. 10.1016/j.tate.2010.07.012

[ref51] Wellington J Ireson G : *Science Learning, Science Teaching.* 3rd ed. Routledge;2012. 10.4324/9780203134962

