# Supplementary material for: Facilitation of practical work in Natural Sciences: experiences and approaches of junior teachers
Source: F1000Res. 2024 Aug 30;13:15. Originally published 2024 Jan 4. [Version 2] doi: 10.12688/f1000research.138137.2 (PMC11525094; doi:10.12688/f1000research.138137.2)
Supplement: Peer review history

# Reviewer report: Reviewer response for version 2

**DOI:** 10.5256/f1000research.169728.r328354

**Published:** 2024-10-30

**Authors:** Neal Petersen

**Affiliations:** 1 North-West University, Potchefstroom, South Africa

I wanted to remind you about the following text:

 This is a timely article addressing perennial issues in science education, such as practical work. The article, titled "Facilitation of practical work in Natural Sciences: experiences and approaches of junior teachers," holds promise in understanding why Natural Sciences teachers do or do not engage in practical work.

 As a general comment, I believe the article shows insufficient logical coherence. This should be addressed.

 Please view the additional review comments in the file found here

Is the work clearly and accurately presented and does it cite the current literature?

Partly

If applicable, is the statistical analysis and its interpretation appropriate?

Not applicable

Are all the source data underlying the results available to ensure full reproducibility?

Yes

Is the study design appropriate and is the work technically sound?

Partly

Are the conclusions drawn adequately supported by the results?

Partly

Are sufficient details of methods and analysis provided to allow replication by others?

Partly

Reviewer Expertise:

Science Teaching; Indigenous knowledge and contextualized learning.

I confirm that I have read this submission and believe that I have an appropriate level of expertise to confirm that it is of an acceptable scientific standard, however I have significant reservations, as outlined above.

# Reviewer report: Reviewer response for version 2

**DOI:** 10.5256/f1000research.169728.r319019

**Published:** 2024-09-17

**Authors:** Tshepo Mokuku

**Affiliations:** 1 National University of Lesotho, Roma, Maseru, Lesotho

Overall the paper has improved, but it is still not satisfactory in terms of coherence. Areas that still require attention include the literature review, theoretical framework and the presentation of findings sections. The literature review still falls short in terms of focus on similar studies; and the section tends to take a rather dogmatic and extended exposition of the policy document (CAPS). What should be pronounced in this section is findings about similar studies elsewhere. The theoretical framework is rather cumbersome, and more complex than required (and applied in the study) I think; it also misses PCK, as the theoretical lens used to answer research question 2. Many parts of the Findings and Discussion section sound rather loose, and the claims made are based on limited evidence (e.g conclusion on the predisposition of the teacher on practice work, based on one lesson observation; albeit not clear as to how many lessons were actually observed): such limited evidence requires somewhat tentative assertions/claims. Perhaps some background information should also be provided in respect of the *nature of questions* the teachers were asked to address the RQs, the *number of practical work lessons* observed per topic, what *informed the choice *of the practical lessons & science topics, and the *connection between* interview questions and practical lessons. Additionally, the findings presented to answer Research Question 1, do not sound well aligned with the research question; some fine tuning is required here; perhaps the researchers could further clarify to the reader, the meaning and significance of the “experiences” they expected the teachers to articulate, in relation to in Research question 1.

Is the work clearly and accurately presented and does it cite the current literature?

Yes

If applicable, is the statistical analysis and its interpretation appropriate?

Not applicable

Are all the source data underlying the results available to ensure full reproducibility?

Yes

Is the study design appropriate and is the work technically sound?

Partly

Are the conclusions drawn adequately supported by the results?

Partly

Are sufficient details of methods and analysis provided to allow replication by others?

Partly

Reviewer Expertise:

NA

I confirm that I have read this submission and believe that I have an appropriate level of expertise to confirm that it is of an acceptable scientific standard, however I have significant reservations, as outlined above.

# Reviewer report: Reviewer response for version 1

**DOI:** 10.5256/f1000research.151314.r240566

**Published:** 2024-03-05

**Authors:** Tshepo Mokuku

**Affiliations:** 1 National University of Lesotho, Roma, Maseru, Lesotho

**The paper title and the abstract**

 First, the title of the paper sounds appropriate, and reflects the gist of the article. The abstract is generally comprehensive, but could be improved by reflecting the question(s) investigated or a clearly articulated purpose, as well as the theoretical framework for the paper, which should also be reflected in the body of the paper. Not all the abstract keywords, sound central to the paper in its present version.

**The Paper as a Whole and its Originality**

 Taken as a whole the article is consistent, and the methodology used sounds appropriate and aligned to the research questions. It sounds original and with further refinement and tightening it could contribute some valuable knowledge to the field.

**Research Questions and Relevant Research, and Theoretical framework**

 The context in which the research questions were formulated is largely clear. The article is, however, limited on relevant literature based on similar studies. Such literature would help inform the discussion of the findings. In addition, the theoretical framework of the study is not distinct and clearly articulated; however, some aspect of the theory that the researchers’ applied in their analysis, seem to surface in the discussion section, and relate to concepts such as ‘explanatory model’ ‘performance model’ ‘procedural knowledge’, and ‘PCK’; these concepts could form part of the theoretical framework section of the study.

**Design and Execution of Methodology**

 The design of the methodology is generally well thought out, but the application could be improved to enhance dependability and interpretation of the findings. This could be achieved by providing detailed descriptions of the practical lessons conducted by individual teachers, based on individual teacher’s written lesson plans; and the lesson plans could constitute part of the documents that the researchers’ analyse. These lesson descriptions could further be enriched by the researchers’ own descriptions of the practical lessons, based on their recorded observation notes. Findings and Discussion The presentation of the results could be improved by generating more detailed textual data, as suggested above, and by analysing the data in terms of distinct themes and patterns, guided by the developed theoretical framework. Several parts of the discussion section are somewhat loose and not well substantiated. This can be corrected by inclusion of the theoretical framework section in the manuscript, to inform/guide the discussion, and by addition of descriptive textual data (in respect of the teachers’ practical lessons), to enhance the dependability and interpretation of the data.

**Conclusion**

 The conclusions need to be tightened further.

**Grammar and Referencing**

 Several typographical or grammatical errors were spotted, and a language focused review of the paper would certainly help improve the clarity of arguments. The researchers are also advised to pay attention to in-text referencing: there are several cases where sources seem to be unnecessarily cited or cited for no apparent reason; and cases where non-authoritative authors on the subject/concept used are acknowledged e.g. The author whose work is not methodology-based, cited against a methodology concept(s).

Is the work clearly and accurately presented and does it cite the current literature?

Yes

If applicable, is the statistical analysis and its interpretation appropriate?

Not applicable

Are all the source data underlying the results available to ensure full reproducibility?

Yes

Is the study design appropriate and is the work technically sound?

Partly

Are the conclusions drawn adequately supported by the results?

Partly

Are sufficient details of methods and analysis provided to allow replication by others?

Partly

Reviewer Expertise:

NA

I confirm that I have read this submission and believe that I have an appropriate level of expertise to confirm that it is of an acceptable scientific standard, however I have significant reservations, as outlined above.

# Response

**Published:** 2024-07-26

**Authors:** Lettah Sikhosana

**Affiliations:** Science & Technology Education, University of South Africa, Muckleneuk, South Africa

Greetings 

 I hope you are doing well. 

 We would like to thank you for reviewing our manuscript. Your comprehensive feedback is highly appreciated. We have gone through the manuscript and the comments that you have made, and we have managed to address the corrections as suggested which have been factored in on the new version of this manuscript. 

 Best wishes

# Reviewer report: Reviewer response for version 1

**DOI:** 10.5256/f1000research.151314.r240562

**Published:** 2024-02-15

**Authors:** Sam Ramaila

**Affiliations:** 1 University of Johannesburg, Johannesburg, South Africa

Clarity of Purpose:

 The introductory statement provides a clear and concise overview of the paper's focus, which is the exploration of junior teachers' experiences and approaches in facilitating practical work in Natural Sciences during teaching and learning.

 Contextual Background:

 The mention of Grade 7 being the final year of primary school in South Africa, along with information about the age group (11 to 13 years), establishes the context for the study. This background information helps readers understand the specific educational setting and the target age group.

 Identification of Challenges:

 The researchers explicitly state that they observed "enormous challenges" faced by junior grade 7 teachers when preparing, designing, and conducting practical work in Natural Sciences. This identification of challenges sets the stage for the research problem and emphasizes the significance of the study.

 Scope and Subject Focus:

 The paper specifies that the focus is on practical work in Natural Sciences, providing clarity about the subject matter under investigation. This clarity is essential for readers to understand the specific context of the study.

 Research Design:

 The choice of a qualitative interpretative case study design is justified, aligning with the research objectives of exploring the experiences and approaches of grade 7 junior teachers in Natural Sciences teaching. The rationale for using an interpretative research paradigm is explained, emphasizing the understanding of lived experiences and perspectives in a social context. The two identified themes provide a clear focus for the investigation.

 Participants:

 The sampling method, purposively selecting three junior grade 7 teachers, is justified based on the aim of obtaining rich and unique data. The criteria for classifying teachers as "junior" (with less than 1 year of teaching experience) is specified. The demographic profile of each teacher is planned for presentation in Table 1, providing a comprehensive overview of the participants.

 Language and Presentation:

 The language used is clear and straightforward, making the information accessible to a broad audience. The sentence structure is well-organized, contributing to the overall readability of the introductory statement.

 Research Gap and Importance:

 While the challenges are highlighted, the introductory statement does not explicitly mention the existing gap in the literature or why addressing these challenges is important. Including a brief discussion on the gap in knowledge or the broader significance of the study could enhance the introduction.

 Conclusion:

 Overall, the introductory statement effectively outlines the purpose, context, and identified challenges of the study. To further strengthen the introduction, the researchers could consider providing a brief rationale for why addressing these challenges is crucial in the field of education or how it contributes to existing knowledge.

Is the work clearly and accurately presented and does it cite the current literature?

Yes

If applicable, is the statistical analysis and its interpretation appropriate?

Not applicable

Are all the source data underlying the results available to ensure full reproducibility?

Yes

Is the study design appropriate and is the work technically sound?

Yes

Are the conclusions drawn adequately supported by the results?

Yes

Are sufficient details of methods and analysis provided to allow replication by others?

Yes

Reviewer Expertise:

Nature of Science, Inquiry-based learning

I confirm that I have read this submission and believe that I have an appropriate level of expertise to confirm that it is of an acceptable scientific standard.

# Reviewer report: Reviewer response for version 1

**DOI:** 10.5256/f1000research.151314.r236948

**Published:** 2024-02-15

**Authors:** Doras Sibanda

**Affiliations:** 1 University of KwaZulu-Natal, Durban, South Africa

Introduction

 Thank you for the opportunity to review the manuscript.  I found the topic to be interesting and relevant. However, the introduction seems to focus more on teachers’ experiences and limited explanation is provided on facilitation of practical work.

 Literature Review

 The literature review is on practical work. There is a need to link the introduction to the problem statement and the title. The authors need to explain why practical work is important in this study.  

 The Research Methods

 The sampling of the seven teachers needs to be clarified. There are three primary schools and does this mean that all the teachers at these schools were participants. How were the teachers recruited? There are too many research questions for the limited data set. There is a need to provide some details about the lessons observed, were the teachers teaching the same topics?

 Data analysis

 There is a need to clarify how the themes were generated. One of the themes is on pedagogical approaches but in the literature review the theoretical framework was not clearly stated. From page 9 paragraph 2, the findings are linked to PCK. There is a need to pride a theoretical background of the study. 

Is the work clearly and accurately presented and does it cite the current literature?

Partly

If applicable, is the statistical analysis and its interpretation appropriate?

Not applicable

Are all the source data underlying the results available to ensure full reproducibility?

Yes

Is the study design appropriate and is the work technically sound?

Partly

Are the conclusions drawn adequately supported by the results?

Partly

Are sufficient details of methods and analysis provided to allow replication by others?

Partly

Reviewer Expertise:

science education, pedagogical content knowledge, mixed methods research

I confirm that I have read this submission and believe that I have an appropriate level of expertise to state that I do not consider it to be of an acceptable scientific standard, for reasons outlined above.
